# Plasma metabolomics and clinical predictors of survival differences in COPD patients

**DOI:** 10.1186/s12931-019-1167-y

**Published:** 2019-10-15

**Authors:** Victor Pinto-Plata, Ciro Casanova, Miguel Divo, Yohannes Tesfaigzi, Vince Calhoun, Jing Sui, Francesca Polverino, Carmen Priolo, Hans Petersen, Juan Pablo de Torres, Jose Maria Marin, Caroline A. Owen, Rebeca Baz, Elizabeth Cordova, Bartolome Celli

**Affiliations:** 1 Pulmonary-Critical Care Medicine, Brigham and Women’s Hospital, Harvard Medical School, Boston, USA; 2 Pulmonary-Critical Care Medicine Division, Baystate Medical Center, University of Massachusetts-Baystate, 759 Chestnut St, Springfield, MA 01199 USA; 3 Servicio de Neumologia, Hospital Universitario Nuestra Señora de la Candelaria, Tenerife, Spain; 4 Lovelace Respiratory Research Institute, Albuquerque, USA; 5 The Mind Research Network, Lovelace Respiratory Research Institute, Albuquerque, USA; 6 Servicio de Neumologia, Clinica Universidad de Navarra, Pamplona, Spain; 7 Servicio de Neumologia, Hospital Universitario Miguel Servet, Zaragoza, Spain

**Keywords:** COPD, Metabolomics, Survival, Dyspnea, Exercise capacity

## Abstract

**Background:**

Plasma metabolomics profile (PMP) in COPD has been associated with clinical characteristics, but PMP’s relationship to survival has not been reported. We determined PMP differences between patients with COPD who died an average of 2 years after enrollment (Non-survivors, NS) compared to those who survived (S) and also with age matched controls (C).

**Methods:**

We studied prospectively 90 patients with severe COPD and 30 controls. NS were divided in discovery and validation cohorts (30 patients each) and the results compared to the PMP of 30 S and C. All participants completed lung function tests, dyspnea scores, quality of life, exercise capacity, BODE index, and plasma metabolomics by liquid and gas chromatography / mass spectometry (LC/MS, LC/MS^2^, GC/MS). Statistically, we used Random Forest Analysis (RFA) and Support Vector Machine (SVM) to determine metabolites that differentiated the 3 groups and compared the ability of metabolites vs. clinical characteristics to classify patients into survivors and non-survivors.

**Results:**

There were 79 metabolites statistically different between S and NS [*p* < 0.05 and false discovery rate (q value) < 0.1]. RFA and SVM classification of COPD survivors and non-survivors had a predicted accuracy of 74 and 85% respectively. Elevation of tricyclic acid cycle intermediates branched amino acids depletion and increase in lactate, fructose and xylonate showed the most relevant differences between S vs. NS suggesting alteration in mitochondrial oxidative energy generation. PMP had similar predictive power for risk of death as information provided by clinical characteristics.

**Conclusions:**

A plasma metabolomic profile characterized by an oxidative energy production difference between survivors and non-survivors was observed in COPD patients 2 years before death.

**Electronic supplementary material:**

The online version of this article (10.1186/s12931-019-1167-y) contains supplementary material, which is available to authorized users.

## Background

Chronic obstructive pulmonary disease (COPD) is a significant cause of morbidity and mortality around the world [1] .The disease is diagnosed by the presence of persistent airflow limitation in subjects with exposure to the appropriate risk (cigarette smoking and/or exposure to environmental pollution) [2] .It has several systemic repercussions and is associated to comorbidities that impact on survival [3]. The severity of airflow limitation and dypsnea and the presence of low body mass index and decrease exercise capacity are known clinical factors able to predict risk of death, especially when integrated into the multidimensional BODE index [4].

Metabolomic profiling refers to the systematic analysis of low molecular weight biochemicals, including sugars, amino acids (AA’s), organic acids, nucleotides and lipids in a biological specimen [5]. In COPD patients, several platforms and matrices have been studied including exhaled gas condensate, urine and plasma [6, 7]. More recently, a plasma metabolic profiling (PMP) has shown association of several amino acids with cachexia and emphysema, as reported in the ECLIPSE cohort [8]. A subsequent study described the relation between 34 targeted amino acids and dipeptides in different subgroups of COPD patients (emphysema, airway disease or cachexia) [9]. In patients from the COPDGene cohort, Bowler and coworkers observed relations between sphingomyelin and ceramides with airflow obstruction and emphysema [10].

We hypothesized that patients with severe COPD at risk of dying have metabolomic alterations that differentiate them from survivors and that the discriminative power of the metabolites would be similar to that provided by the clinical information provided by the BODE index and its variables. To test this hypothesis, we performed an untargeted metabolomic profiling of COPD patients and age matched controls, that included not only amino acids, but also peptides, carbohydrates, components of the Krebs cycle, oxidative phosphorylation, and several lipids (essential, medium and long chain fatty acids, lyso and sphingolipids) to describe a more global metabolic disarrangement that could differentiate survivors from non-survivors.

## Materials and methods

### Clinical data

We prospectively recruited and followed for over 3 years a group of 90 COPD patients and 30 age-gender matched controls (C). The 60 COPD patients that died during the study (non-survivor or NS) were equally divided in 2 groups: discovery (NSd) and validation (NSv). The NSd group was compared to the survivor (S) COPD group (*n* = 30) and a control group (n = 30 volunteers with no lung disease, 40% current smokers and 60% non- smokers). Results from the 30 NSv were used to validate the findings of this analysis (Fig. 1 in the supplement). The mean (SD) survival time for NS was 24 + 19 months. All patients were recruited in Boston and Tenerife (NSv only) following the same protocol approved by the IRB at both institutions. Lung function, 6 min walk test (6MWT), dyspnea level (modified Medical Research Council, mMRC scale), the Saint George Respiratory Questionnaire (SGRQ) and the BODE index [4] were measured at baseline. Blood was drawn early morning under fasting condition.

**Fig. 1:**
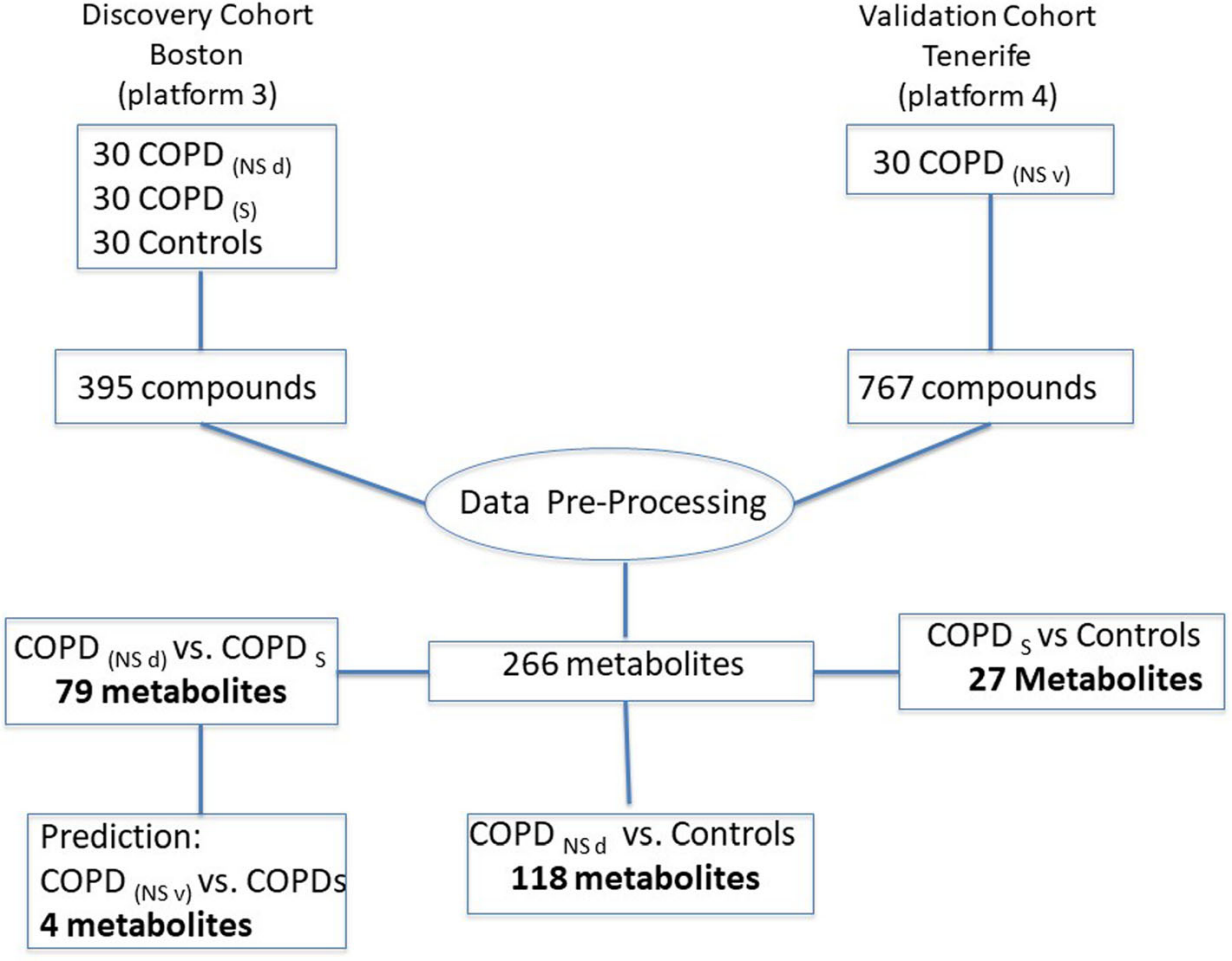
Flow diagram describes the discovery and validation cohorts, data processing and number of metabolites determined on each experiment

### Plasma metabolomic analysis

Plasma metabolomic profile was determined by 2 platforms (version 3 and 4 as described in Fig. 1), using Liquid Chromatography/ Mass Spectometry (LC/MS, LC/MS^2^) and Gas Chromatography/ Mass Spectrometry (GC/MS). Both analyses were completed at Metabolon, Inc. (Durham, NC) (details on the supplemental material (SM) files). Metabolites identified in 80% of both platforms were included in the final analysis, resulting in the inclusion of 266 metabolites.

### Statistical methods

We performed a t test and analysis of variance (ANOVA) with Bonferroni correction for multiple comparisons for the clinical data among groups.

We used log transformation and imputation with minimum observed values for each metabolite. Then, we analyzed the data using 3 complementary methods. First, Welch’s two-sample *t*-test to identify biochemicals that differed significantly between the COPD groups (NSd and S) and control population (C). A list of metabolites that differentiate each group was generated. The false discovery rate (FDR) q value was used to correct for multiple comparisons.

Second, for group classification, we completed two different analyses; Random Forest (RF) and Support Vector Machine (SVM) [11] They both estimate how well we can classify individuals in a new data set into each group. Random Forest generates a set of classification trees based on continual sampling of the experimental units and compounds. Then each observation is classified based on the majority votes from all the classification trees [12]. The SVM identifies a linear, maximal-margin decision boundary between the sample groups by solving a quadratic optimization problem [13]. We used both methods to increase the validity of the group classification results.

Third, we used a two-sample t-test, linear discriminate analysis (LDA) and SVM-RFE (a recursive feature elimination or SVM-RFE) to determine how well clinical characteristics and metabolites classify patients into 3 different groups (COPD survivors, non-survivors and controls). (See SM). Finally, we used the list of metabolites that separated survivors versus non-survivors discovery to predict and validate the comparison of NSv group vs. S and to perform a pathways analysis using MetaboAnalyst software [14].

## Results

The clinical characteristics of each group are included in Table 1. The subjects in the four groups were similar in age, gender, body mass index, comorbidities and smoking history. As expected, COPD survivors had better lung function, exercise capacity, quality of life, gas exchange and lower BODE index than non-survivors, while controls had normal lung function, with no difference between smokers and non-smokers.

**Table 1:** Clinical characteristics of COPD patients and control population

Variables	COPD (NS d)	COPD (NS v)	COPD (S)	Controls	*p* value
N	30	30	30	30	
Age (y)	67 ± 10	71 ± 8	68 ± 7	68 ± 7	0.72
Male, %	68	70	59	67	0.73
BMI (kg/m^2^)	25.8 ± 5	26.7 ± 5	27.3 ± 4	28.8 ± 5	0.05
Smoking (p/y)	68 ± 36	65 ± 6	60 ± 38	58 ± 30	0.5
FVC, %	71 ± 26	70 ± 18	79 ± 14	104 ± 16	< 0.001
FEV1, %	35 ± 12	46 ± 20	45 ± 15	99 ± 17	< 0.001
FRC, %	181 ± 48	153 ± 46	155 ± 47	97 ± 24	< 0.001
DLCO, %	44 ± 14	56 ± 23	64 ± 22	91 ± 24	< 0.001
IC/TLC	0.22 ± 0.06	0.27 ± 0.08	0.33 ± 0.12	0.47 ± 0.09	< 0.001
mMRC	2.7 ± 0.6	1.7 ± 1.4	1.5 ± 1.1	0.13 ± 0.3	< 0.001
6MWT(m)	301 ± 92	408 ± 77	442 ± 125	543 ± 113	< 0.001
BODE	5.3 ± 1.6	3.1 ± 2.2	3 ± 1.9	N/A	< 0.001
GOLD 2,3,4, %	6,52,42	40,37,23	34,52,14	N/A	< 0.001
SGRQ	56 ± 15	50 ± 12	42 ± 25	11 ± 13	< 0.001
Charlson CI	0.8 ± 1.2	0.6 ± 1.2	0.5 ± 1.1	0.3 ± 0.7	0.30
PaO_2_ (mmHg)	65 ± 14	58 ± 10	76 ± 11	N/A	< 0.002
PaCO_2_ (mmHg)	46 ± 9	45 ± 6	40 ± 3	N/A	< 0.002

*NS d* Non-survivor discovery. *NS v* Non-survivor validation *S* Survivors. *Charlson CI* Charlson Comorbidity Index. *N/A* Non-applicable

### Metabolites identification

The 2 platforms measured a total of 395 and 767 compounds of known identity (Fig. 1). Two hundred and sixty-six metabolites were identified and included in the final analysis.

First, we compared 3 groups (NSd, S and C) and generated a list of metabolites that had achieved statistical significance (*p* ≤ 0.05) among these 3 groups. Results are shown in the SM (Additional file 1: Table S1-S3). Table 2 shows the number of metabolites that were statistically different (t test) in the above-mentioned analysis. There were 108, 79 and 27 significantly altered biochemicals (*p* ≤ 0.05) between NS vs. C, NS vs. S and S vs. C, suggesting a larger metabolic disarrangement as the patients were clinically more compromised.

**Table 2:** Comparison between the 3 different groups. Biochemicals in red are elevated and in green are reduced

Altered biochemicals	NSd vs. C	NSd vs. S	S vs. C
Total Biochemicals (*p* < 0.05)	108	79	27
Biochemicals	68/40	54/25	7/20
Total Biochemical(0.05 > *p* < 0.1)	21	9	19
Biochemicals	14/7	6/3	11/8

Number of elevated () or reduced () metabolites in the group comparison

**Fig. 2:**
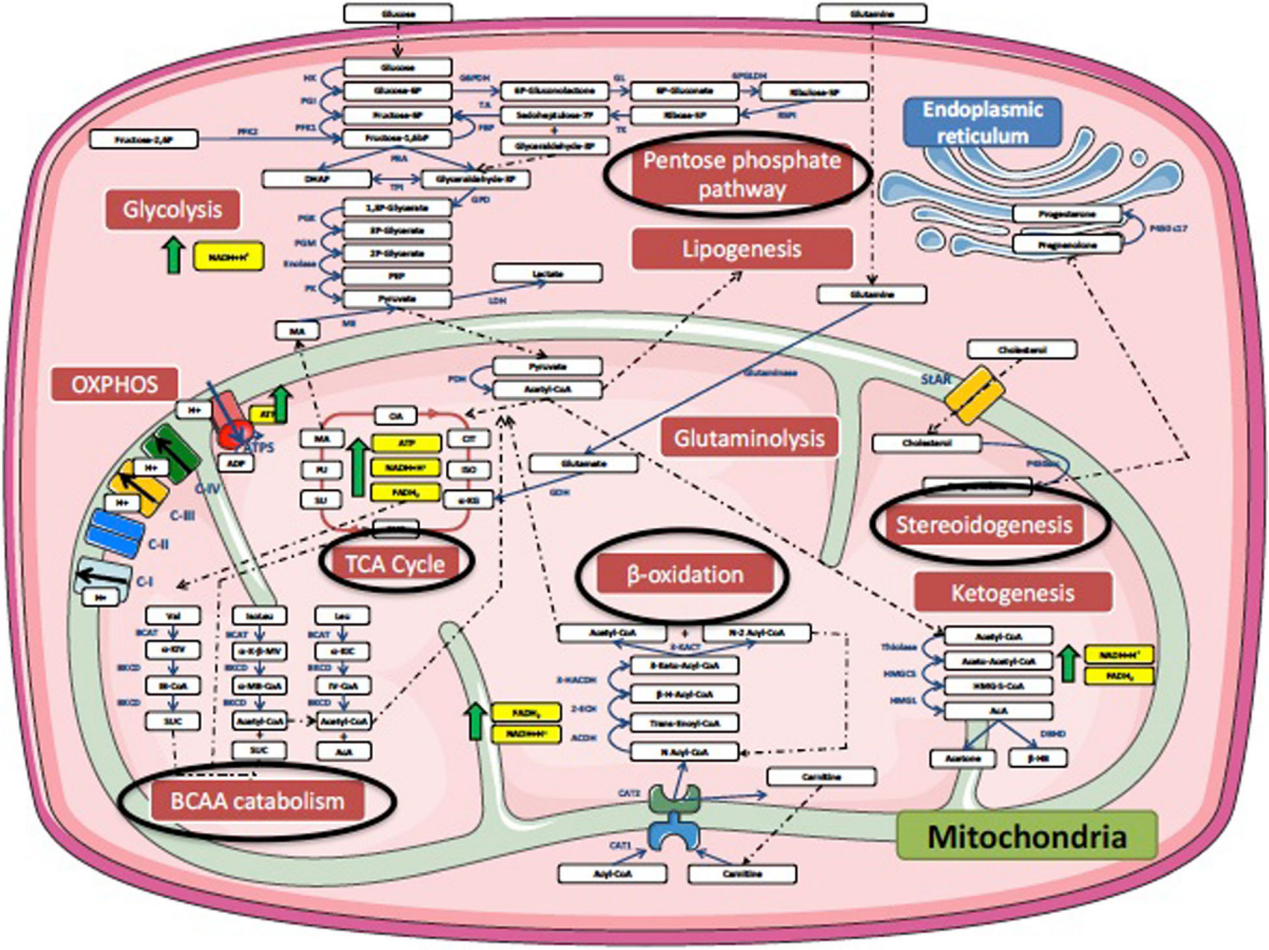
Cell diagram depicting metabolomic differences between COPD non-survivors (NS) and COPD survivors (S). The black circles denote altered metabolic pathways. The majority of these pathways were related to mitochondrial function

There were pronounced elevations in the Tricarboxylic Acid (TCA) cycle intermediates alpha-ketoglutarate, succinate/succinylcarnitine, succinate, fumarate, and malate in COPD non-survivor discovery. Reductions in circulating levels of the branched-chain amino acids (BCAAs) leucine, isoleucine, and valine were noted in COPD non-survivors, along with increased lactate, fructose, and five-carbon sugars alcohols such as xylonate that are produced through the pentose phosphate pathway (PPP). Figure 2 shows a cell diagram of the metabolomic alterations, most of which are found within the mitochondria. Similar findings were observed in the non-survivors in the validating group (NSd).

### Group classification by metabolites

The RF and SVM analyses were used to assess the separability of the sample into groups and rank metabolites that are significantly different between groups. (Table 3). The RF and SVM suggested a better discrimination between the NSd and C since the misclassification rate was the lowest for this 2-groups comparison. The ranking classification of the metabolites in each group was similar irrespective of the statistical method used, supporting the validity of the results.

**Table 3:** Results of 2 different group classification strategies (SVM and RFA) using statistically significant metabolites (*p* < 0.05). All values reported as misclassification rates (lower is better). A rate of approximately 0.5 is equal to chance alone. NSd: non-survivors discovery group. S: Survivor group C: Control group

Groups	Support Vector Machine (SVM)	Random Forest Analysis (RFA)
NSd. vs. S	0.33	0.30
NSd vs. C	0.18	0.14
S vs. C	0.37	0.37

### Group classification using metabolites vs. clinical results

**Fig. 3:**
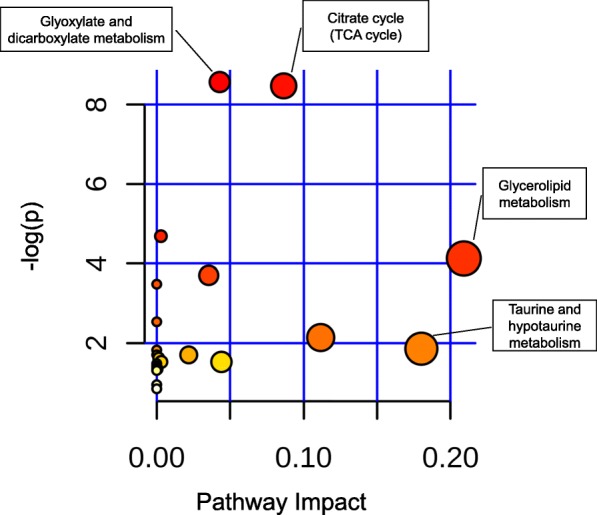
Metabolome overview depicting matched pathways according to *p* values from the pathway enrichment analysis (vertical axis) and the pathway impact values from the pathway topology analysis (horizontal axis) [15]. The most significant and over-represented pathways are related to energy metabolism

The capacity of the metabolites to classify patients in different groups compared to the combination of clinical data (BODE index)) is shown in Table 4. This analysis demonstrated that a smaller group of metabolites (26, 12 and19) could differentiate and classify patients in each group with excellent accuracy (0.73–0.78) (Additional file 1: Table S4). The 26 metabolites classified patients in the 2 COPD groups (Survivors and non-survivors) with remarkable similarity to the clinical data. We performed a pathway analysis [14] with these 26 metabolites that confirmed metabolic pathway alterations in TCA cycle but also in glyoxylate and dicarboxylate and glycerolipid metabolism (Fig. 3).

**Table 4:** Results of the sensitivity, specificity and accuracy analysis to determine the capacity of the list of metabolites (1st digit) and clinical data (2nd digit) to classify patients in each group. NSd: COPD non-survivor discovery. S: COPD survivor. C: Control

Group Comparison	Sensitivity		Specificity		Accuracy	
	Metabolites	Clinical	Metabolites	Clinical	Metabolites	Clinical
NSd vs. S	0.79	0.73	0.77	0.81	0.78	0.78
NSd vs. C	0.8	1	0.77	1	0.79	1
S vs. C	0.8	0.96	0.66	0.89	0.73	0.93

### Survival prediction

After elimination of xenobiotics from the initial list of 266 metabolites, 244 biochemicals were used to determine a final list of biochemicals that related to survival. The metabolite list generated during discovery (NSd vs. S) was compared for validation to NSv vs. S. We used SVM and t test analysis to rank by weight metabolites that predicted survival. Four metabolites: fructose, hexadecadenionate, hwesasxx (a fibrinogen split product) and oxalate predicted survival with 85% accuracy, 81% sensitivity and 89% sensitivity (Fig. 1).

## Discussion

This study reports three novel findings. First, there are differences in plasma metabolomics profile between COPD patients who die over a period of 3 years and those that do not. Secondly, the differences in metabolite between groups allowed the correct classification of patients based on their metabolomic signature alone. This signature validated in a different cohort was as good as several clinical parameters combined in differentiating survivors from non survivors. Lastly, metabolic pathways in COPD survivors and non-survivors identified several disruptions, particularly in energy metabolism, suggesting that mitochondrial dysfunction plays an important role in the non-survival group.

Previous studies have reported association of metabolic signature of patients with COPD [8, 10, 16–18], with clinical characteristics and outcomes including degree of airway obstruction and severity of emphysema, exacerbation and hospitalization but only one has evaluated survival differences [19]. However, in that study the analysis was limited to adrenal hormone metabolites. Different biologic matrices (plasma, urine and exhaled condensates) [20, 21] and data sets of functional genomics, proteomics and metabolomics have also been published revealing the complexity of true “integrated omics [22] Previous work by Ubhi and colleagues, found differences between COPD patients and control subjects, and COPD patients characteristics (disease severity and phenotypes) [8]. The authors specifically validated amino acids and dipeptides and correlated them to cachexia, emphysema and GOLD 4 category [9]. A more recent study by Bowler and colleagues [10] described the association between plasma sphingolipids and sub-phenotypes of COPD in over 250 patients from the COPDGene cohort. Five sphingomyelins were associated with emphysema and 7 ceramides with COPD exacerbation. This group also described a 15 sphingolipids gene/metabolite pairs differentially regulated between COPD and control subjects. Despite differences in study design, number of subjects, and quantification methods, our findings were similar to the ones reported in these 2 cohorts, including a reduction in 3 amino acids (branched-chain) and increase in 2 sphingomyelins (palmitoyl and stearoyl sphingomyelin) in COPD compared to control subjects.

Our study expands the current knowledge by reporting a larger number of metabolites albeit in a smaller number of subjects than previously reported and relating the difference to risk of death (Table 2). We confirm large metabolic differences between COPD patients and controls but also between COPD patients who died versus those that survived over 3 years of observation. This differences between patients with different disease severity is consistent with the findings by Ubhi et al. who showed significant differences in metabolomic profile between patients with GOLD stages III and IV vs. controls without COPD, but not between GOLD II patients and controls [8]. This observation further supports the notion that the metabolite signature can classify patients in 3 different groups. As shown in Table 3, we used 2 different supervised learning algorithms to assess the predictive power of the metabolites to classify subjects according to 3 different groups. A better classification capacity was noted by the 2 methods when differentiating COPD non-survivors vs. controls compared to the other sets of classification (NSd vs. S and S vs. C). Nevertheless, the similarity of the results using both methods supports the validity of the observations.

To further study the ability of the metabolomic analysis as a classification tool, we compared the appropriateness of patient allocation into the 3 groups by metabolic data and a comprehensive panel of clinical parameters. The clinical parameters selected included several with demonstrated capacity to predict survival, either individually (FEV_1_, dyspnea level, distance walked, BMI, lung volumes, Pa CO_2_) or the combination of them (BODE index). The classification for the COPD patients (survivors and non-survivors) was quite similar using both strategies (Table 4). This approach could be particularly useful since patients in the survivor and non-survivor groups had severe disease and predicting survival for any clinician is difficult. Even though we validated the results in a subsequent group of patients with COPD, further validation in other cohorts is needed to solidify for the possible use of metabolomic signatures as prognostic tools in “personalized” medicine.

### Metabolomic pathways in COPD survivors and non-survivors

Figures 2 and 3 and Additional file 1: Table S5 show differences in metabolomic pathways comparing COPD survivors vs. non-survivors. Significant alterations were observed in the in the TCA cycle with accumulation of several intermediaries (alpha-ketoglutarate, succinate, fumarate, malate), in biochemicals related to glucose metabolism including elevation in lactate, glycerate, fructose, in the pentose phosphate pathway with accumulation of five sugar carbon alcohol xylonate and other sugar alcohols threitol, arabitol and fucose and the glyoxylate and dicarboxylate metabolism as well as the glycerolipid pathway.

These abnormalities in association with a reduction in levels of branched-chain amino acids valine, leucine, isoleucine, while not confirmed in the validation group, suggests an altered state of oxidative stress. Green et al. [23] has suggested the existence of “metabolic check points” that determine cell death; including acetyl CoA production abnormalities involving TCA and pentose phosphate pathway as well as an increase in the production of sphyngolipids. Our results in peripheral blood, could represent a reflection of these alterations in cells throughout the body.

Besides energy metabolism, we found an increase in circulating levels of polypeptides associated to fibrinogen cleavage in the non-survivor group compared to the survivors (confirmed in the validation group) and the biologically-active bradykinin metabolite bradykinin, des-arg-9. Bradykinin is associated to activation of Factor Xll and fibrinogen has been associated to increased risk of exacerbation and all-cause mortality in COPD [24, 25].

To our knowledge, this is the first study in COPD patients where a metabolomic signature based on a wide range of metabolites has been associated with mortality. However, metabolomic analysis have been used to predict clinical outcome in other conditions, including sepsis [26, 27], recurrent breast cancer [28] and heart failure post intervention [29].

This manuscript has several limitations. First, a relatively small number of subjects were enrolled in this study, particularly when comparing to previous work by Ubhi et al. [8] and Bowler and coworkers [10]. A recent article on biomarker discovery suggested a large sample size (> 500 patients) and reproducibility in at least one external cohort [30]. To overcome this limitation, we included a confirmatory validating cohort and performed several independent statistical analyses completed by researchers blinded to each other. Indeed, the classification according to groups (COPD survivors, non-survivors and control) was tested using 2 different statistical analysis (SVM and RFA), both showing similar results. Importantly, we also tested its classification strength against a group of comprehensive clinical parameters and found metabolites to be as good as the panel of clinical characteristic with accuracy over 0.7 (Table 4), a performance benchmark suggested to move biomarker panels into replication and validation phases [30]. We implemented this to strengthen the validity and address the issue of a number of covariates (metabolites) larger than the number of samples (patients) or “p > n problem”. In addition, we used the most widely accepted methods to classify metabolomic data, Random Forest Analysis and Support Vector Machine [11]. Strong support to our findings is provided by the fact that several metabolites in our study were common to previous studies despite differences in patient selection criteria, study design and analysis platform. Second: Two different platforms with different capacity to identify metabolites were used. The confirmatory cohort samples were analyzed using the second a platform (Fig. 1) which may have explained why certain findings (branched-chain amino acids) were not confirmed in the validation cohort. We restricted the selection of metabolites to those identified in 80% of the samples from both platforms, limiting the number of metabolites identified but increasing the validity of the results. This may have also reduced the number of common metabolites found on the verification cohort to only 4. A third limitation is inherent to metabolomic studies in general and refer to the lack of a clear or unique metabolic signature for each disease and lack of power analysis and sample size estimation. The relatively small sample size may have limited the number of metabolites and pathways alterations associated to increased mortality. However, the different and complementary statistical analysis showing significant differences in several metabolites and pathways provide support to our findings. That the clinical data and metabolomic results have a similar prediction for survival is encouraging. In addition, pathways shown in KEGG charts capture about 90% of the chemical mass but only show about 60% of the total number of pathways. However, given that this field is in constant evolution, we believe that our findings will help stimulate more research in this area.

A potential fourth limitation is the composition of the control group, including both smokers and non-smokers. Although the study was designed to determine differences in survival in the COPD group, the lung function was normal and similar between the smokers and non-smokers and the smoking history in the former was similar to that of COPD patients thereby minimizing the role of smoking on the findings.

## Conclusion

We have shown that plasma metabolomic profile differs between COPD patients and controls, with more divergence as the disease is more severe. The findings offer evidence that several pathways are involved including energy metabolism, probably associated to oxygen transport and mitochondrial dysfunction, and the coagulation cascade. The metabolic signature closely matches the ability of clinical characteristics to classify patients with COPD as survivors and non -survivors. On average, samples were drawn 2 years prior to patient’s death, suggesting that plasma metabolomics could have a place in the clinical management of patients with severe disease and may help not only to predict outcome but maybe useful as a tool for intervention and as markers of response to treatment.

## Additional file

Additional file 1: Plasma Metabolic Profile and COPD Survival. (DOCX 69 kb)

## Data Availability

All data generated or analyzed during this study are included in this published article and its supplementary information files.

## Abbreviations

6 MWD: Six Minute Walk Distance
BCAAs: Branched-chain amino acids
BODE: Body mass index, Obstruction. Dyspnea. Exercise
COPD: Chronic Obstructive Pulmonary Disease
FDR: False Discovery Rate
GC: Gas Chromatography
LC: Liquid Chromatography
mMRC: modified Medical Research Council
MS: Mass Spectrometry
PMP: Plasma Metabolomic Profile
RFA: Random Forrest Analysis
SGRQ: Saint George Respiratory Questionnaire
SVM: Support Vector Machine
TCA: Tricarboxylic acid

## References

[1] Buist AS, Vollmer WM, McBurnie MA (2008). Worldwide burden of COPD in high- and low-income countries. Part I. The burden of obstructive lung disease (BOLD) initiative. Int J Tuberc Lung Dis.

[2] Celli BR, MacNee W, Agusti A, Anzueto A, Berg B, Buist AS (2004). Standards for the diagnosis and treatment of patients with COPD: A summary of the ATS/ERS position paper. Eur Respir J.

[3] Divo M, Cote C, De Torres JP, Casanova C, Marin JM, Pinto-Plata V (2012). Comorbidities and risk of mortality in patients with chronic obstructive pulmonary disease. Am J Respir Crit Care Med.

[4] Celli BR, Cote CG, Marin JM, Casanova C, Montes de Oca M, Mendez RA (2004). The body-mass index, airflow obstruction, dyspnea, and exercise capacity index in chronic obstructive pulmonary disease. N Engl J Med.

[5] Psychogios N, Hau DD, Peng J, Guo AC, Mandal R, Bouatra S (2011). The human serum metabolome. PLoS One.

[6] Fens N, De Nijs SB, Peters S, Dekker T, Knobel HH, Vink TJ (2011). Exhaled air molecular profiling in relation to inflammatory subtype and activity in COPD. Eur Respir J.

[7] Izquierdo-García JL, Peces-Barba G, Heili S, Diaz R, Want E, Ruiz-Cabello J (2011). Is NMR-based metabolomic analysis of exhaled breath condensate accurate?. Eur Respir J.

[8] Ubhi BK, Riley JH, Shaw PA, Lomas DA, Tal-Singers R, MacNeef W (2012). Metabolic profiling detects biomarkers of protein degradation in COPD patients. Eur Respir J.

[9] Ubhi BK, Cheng KK, Dong J, Janowitz T, Jodrell D, Tal-Singer R (2012). Targeted metabolomics identifies perturbations in amino acid metabolism that sub-classify patients with COPD. Mol Biosyst.

[10] Bowler RP, Jacobson S, Cruickshank C, Hughes GJ, Siska C, Ory DS (2015). Plasma sphingolipids associated with chronic obstructive pulmonary disease phenotypes. Am J Respir Crit Care Med.

[11] Korman A, Oh A, Raskind A, Banks D (2012). Statistical methods in metabolomics. Methods Mol Biol.

[12] Chen T, Cao Y, Zhang Y, Liu J, Bao Y, Wang C (2013). Random forest in clinical metabolomics for phenotypic discrimination and biomarker selection. Evid Based Complement Alternat Med.

[13] Byvatov E, Schneider G (2003). Support vector machine applications in bioinformatics. Appl Bioinforma.

[14] Xia J, Sinelnikov IV, Han B, Wishart DS (2015). MetaboAnalyst 3.0-making metabolomics more meaningful. Nucleic Acids Res..

[15] Xia J, Psychogios N, Young N, Wishart DS (2009). MetaboAnalyst: A web server for metabolomic data analysis and interpretation. Nucleic Acids Res.

[16] Bahr TM, Hughes GJ, Armstrong M, Reisdorph R, Coldren CD, Edwards MG (2013). Peripheral blood mononuclear cell gene expression in chronic obstructive pulmonary disease. Am J Respir Cell Mol Biol.

[17] Paige M, Burdick MD, Kim S, Xu J, Lee JK, Michael SY (2011). Pilot analysis of the plasma metabolite profiles associated with emphysematous chronic obstructive pulmonary disease phenotype. Biochem Biophys Res Commun.

[18] Chen Q, Deeb RS, Ma Y, Staudt MR, Crystal RG, Gross SS (2015). Serum metabolite biomarkers discriminate healthy smokers from COPD smokers. PLoS One.

[19] Zurfluh S, Nickler M, Ottiger M, Steuer C, Kutz A, Christ-Crain M (2018). Association of adrenal hormone metabolites and mortality over a 6-year follow-up in COPD patients with acute exacerbation. Clin Chem Lab Med.

[20] Shih YM, Cooke MS, Pan CH, Chao MR, Hu CW (2019). Clinical relevance of guanine-derived urinary biomarkers of oxidative stress, determined by LC-MS/MS. Redox Biol.

[21] Montuschi P, Santini G, Mores N, Vignoli A, Macagno F, Shoreh R (2018). Breathomics for assessing the effects of treatment and withdrawal with inhaled beclomethasone/formoterol in patients with COPD. Front Pharmacol.

[22] Cruickshank-Quinn CI, Jacobson S, Hughes G, Powell RL, Petrache I, Kechris K (2018). Metabolomics and transcriptomics pathway approach reveals outcome-specific perturbations in COPD. Sci Rep.

[23] Green DR, Galluzzi L, Kroemer G (2014). Metabolic control of cell death. Science (80- ).

[24] Duvoix A, Dickens J, Haq I, Mannino D, Miller B, Tal-Singer R, et al. Blood fibrinogen as a biomarker of chronic obstructive pulmonary disease. Thorax. 2012;68:670–6. 10.1164/rccm.201509-1722PP. 10.1136/thoraxjnl-2012-201871 PMC3711372 22744884

[25] Miller BE, Tal-Singer R, Rennard SI, Furtwaengler A, Leidy N, Lowings M, et al. Plasma fibrinogen qualification as a drug development tool in chronic obstructive pulmonary disease. perspective of the chronic obstructive pulmonary disease biomarker qualification consortium. Am J Respir Crit Care Med. 2016;193:607–613. Available from: 10.1164/rccm.201509-1722PP%5Cnhttp://www.ncbi.nlm.nih.gov/pubmed/26745765 10.1164/rccm.201509-1722PP 26745765

[26] Mickiewicz B, Vogel HJ, Wong HR, Winston BW (2013). Metabolomics as a novel approach for early diagnosis of pediatric septic shock and its mortality. Am J Respir Crit Care Med.

[27] Langley RJ, Tsalik EL, van Velkinburgh JC, Glickman SW, Rice BJ, Wang C, et al. An integrated clinico-metabolomic model improves prediction of death in sepsis. Sci Transl Med. 2013;5:195ra95. Available from: https://www.ncbi.nlm.nih.gov/pubmed/23884467. 10.1126/scitranslmed.3005893 PMC3924586 23884467

[28] Asiago VM, Alvarado LZ, Shanaiah N, Gowda GAN, Owusu-Sarfo K, Ballas RA (2010). Early detection of recurrent breast cancer using metabolite profiling. Cancer Res.

[29] Ahmad T, Kelly JP, McGarrah RW, Hellkamp AS, Fiuzat M, Testani JM (2016). Prognostic implications of long-chain acylcarnitines in heart failure and reversibility with mechanical circulatory support. J Am Coll Cardiol.

[30] Sin DD, Hollander Z, DeMarco ML, McManus BM, Ng RT (2015). Biomarker development for chronic obstructive pulmonary disease from discovery to clinical implementation. Am J Respir Crit Care Med.

