# Supplementary material for: Plasma metabolomics and clinical predictors of survival differences in COPD patients
Source: Respir Res. 2019 Oct 15;20:219. doi: 10.1186/s12931-019-1167-y (PMC6794856; doi:10.1186/s12931-019-1167-y)
Supplement: Supplementary file 1 — Plasma Metabolic Profile and COPD Survival. (DOCX 69 kb) [file 12931_2019_1167_MOESM1_ESM.docx]

**Supplemental Material**

**Plasma Metabolic Profile and COPD Survival.**

Victor Pinto-Plata^1,7^, Ciro Casanova ^2^, Miguel Divo^1^, Yohannes Tesfaigzi^3^, Vince Calhoun^4^, Jing Sui ^4^, Francesca Polverino ^1^ , Carmen Priolo^1^, , Hans Petersen ^2^, Juan Pablo de Torres^5^, Jose Maria Marin^6^, Caroline A Owen^1^, Rebeca Baz^2^, Elizabeth Cordova^2^, Bartolome Celli^1^.

**Material and Methods**

**Clinical data:** The study was approved by the IRB at both institutions (St Elizabeth’s Medical Center, Boston, MA, USA study #0299GWL0 and Hospital Universitario Nuestra Señora de la Candelaria, Tenerife, Spain IRB# 258/2009 )

**Sample preparation for measurement of plasma metabolomics:** The sample preparation was carried out using the automated MicroLab STAR® system from Hamilton Company. Recovery standards were added prior to the first step in the extraction process for QC purposes. Sample preparation was conducted using a proprietary series of organic and aqueous extractions to remove the protein fraction while allowing maximum recovery of small molecules. The resulting extract was divided into two fractions; one for analysis by LC and one for analysis by GC. Samples were placed briefly on a TurboVap® (Zymark) to remove the organic solvent. Each sample was then frozen and dried under vacuum. Samples were then prepared for the appropriate instrument, either LC/MS or GC/MS.

**Liquid chromatography/Mass Spectrometry (LC/MS, LC/MS2):** The LC/MS portion of the platform was based on a Waters ACQUITY UPLC and a Thermo-Finnigan LTQ mass spectrometer, which consisted of an electrospray ionization (ESI) source and linear ion-trap (LIT) mass analyzer. The sample extract was split into two aliquots, dried, then reconstituted in acidic or basic LC-compatible solvents, each of which contained 11 or more injection standards at fixed concentrations. One aliquot was analyzed using acidic positive ion optimized conditions and the other using basic negative ion optimized conditions in two independent injections using separate dedicated columns. Extracts reconstituted in acidic conditions were gradient eluted using water and methanol both containing 0.1% Formic acid, while the basic extracts, which also used water/methanol, contained 6.5mM Ammonium Bicarbonate. The Mass Spectrometry(MS) analysis alternated between MS and data-dependent MS2 scans using dynamic exclusion.

**Gas chromatography/Mass Spectrometry (GC/MS):** The samples destined for GC/MS analysis were re-dried under vacuum desiccation for a minimum of 24 hours prior to being derivatized under dried nitrogen using bistrimethyl-silyl-triflouroacetamide (BSTFA). The GC column was 5% phenyl and the temperature ramp is from 40° to 300° C in a 16 minute period. Samples were analyzed on a Thermo-Finnigan Trace DSQ fast-scanning single-quadrupole mass spectrometer using electron impact ionization. The instrument was tuned and calibrated for mass resolution and mass accuracy on a daily basis. The information output from the raw data files was automatically extracted as discussed below.

**Accurate Mass Determination and MS/MS fragmentation (LC/MS), (LC/MS/MS):** The LC/MS portion of the platform was based on a Waters ACQUITY UPLC and a Thermo-Finnigan LTQ-FT mass spectrometer, which had a linear ion-trap (LIT) front end and a Fourier transform ion cyclotron resonance (FT-ICR) mass spectrometer backend. For ions with counts greater than 2 million, an accurate mass measurement could be performed. Accurate mass measurements could be made on the parent ion as well as fragments. The typical mass error was less than 5 ppm. Ions with less than two million counts require a greater amount of effort to characterize. Fragmentation spectra (MS/MS) were typically generated in data dependent manner, but if necessary, targeted MS/MS could be employed, such as in the case of lower level signals.

**Statistical Analysis**

**Random Forest Analysis (RFA)**: It is a supervised classification technique based on an ensemble of decision trees[1,2] . A random subset of the data with identifying true class information is selected to build the tree (“bootstrap sample” or “training set”), and then the remaining data, the “out-of-bag” (OOB) variables, are passed down the tree to obtain a class prediction for each sample.  This process is repeated thousands of times to produce the forest.  The final classification of each sample is determined by computing the class prediction frequency (“votes”) for the OOB variables over the whole forest.

**Support Vector Machine:** A support vector machine is a supervised learning model with associated learning algorithms that analyze data and recognize patterns, used for classification [3]. The basic SVM takes a set of input data and predicts, for each given input, which of two possible classes forms the output, making it a non-probabilistic binary linear classifier. Both RF and SVM models were built using MATLAB (MATLAB version 2012b. Natick, Massachusetts: The MathWorks Inc., 2012).

We implemented a sequential forward selection method (SVM-SFS) and a recursive feature elimination (SVM-RFE).

Sequential forward selection method (SVM-SFS): the process proceeds by creating and cross-validating a model for each metabolite individually (here, 395 SVMs, one for each metabolite). The metabolite yielding the smallest misclassification rate is selected and used in combination with each remaining metabolite to create new cross-validated models, each containing two metabolites. The process repeats until a sufficient number of metabolites are ranked. Due to computational intensity, we ranked the top 20 metabolites for each sample comparison.

We trained SVM using recursive feature elimination (SVM-RFE), which is able to determine a value of each feature, i.e. frequency values, by training a SVM using training samples with class labels to identify a determinative subset. The frequency values ranged from 0 to 1; the higher the value, the more relevant a particular metabolite is for the classification. One or more features having the smallest values are removed and an updated kernel matrix is generated using the remaining features. The process is repeated until a predetermined number of features remain which are capable of accurately separating the data into different classes. SVM-RFE can be used for both feature selection and classification.

Here for the feature selection, we combined three methods: two sample t-test, linear discriminate analysis (LDA) and SVM-RFE. First, features of interest were selected by each of the three methods. Then all of them were combined and trained by SVM-RFE again, by 10-fold cross-validation (trained on 90% of the randomly chosen data samples and tested on the other 10%) for 100 times, finally those features with frequency values (to determine the capacity of metabolites or clinical data to classify patients in each clinical group) bigger than 0.5 were selected. We also recorded the specificity, sensitivity, and frequency values of each feature. This analysis was independently performed by VC and JS.

**Results**

e-Table1-3 Heat Map of the significantly different metabolites between groups.

Color codes:

e-Table1 (Supplemental Material ) List of metabolites that differentiate COPD non-survivor discovery (NS d) vs. Control (C) group. Results are ranked according to the Welch’s t test results. Color coded results according to the level of significance (see legend above)

| **Metabolite** | **NSd / C** | **p value** | **q value** |
| --- | --- | --- | --- |
| 2-ethylhexanoate | **6.85** | 0.0000 | 0.0000 |
| Benzoate | **0.62** | 0.0000 | 0.0000 |
| bradykinin, des-arg(9) | **5.63** | 0.0000 | 0.0000 |
| Hexadecanedioate | **2.35** | 0.0000 | 0.0000 |
| Fucose | **1.98** | 0.0000 | 0.0000 |
| HWESASXX* | **2.23** | 0.0000 | 0.0000 |
| Malate | **2.02** | 0.0000 | 0.0000 |
| 2-aminobutyrate | **0.75** | 0.0000 | 0.0000 |
| 1-arachidonoylglycerophosphoinositol* | **1.55** | 0.0000 | 0.0000 |
| dihomo-linolenate (20:3n3 or n6) | **1.63** | 0.0000 | 0.0000 |
| Succinate | **1.51** | 0.0000 | 0.0000 |
| Fumarate | **1.45** | 0.0000 | 0.0001 |
| Lactate | **1.38** | 0.0000 | 0.0001 |
| Aspartate | **1.93** | 0.0000 | 0.0001 |
| Ornithine | **1.83** | 0.0000 | 0.0001 |
| 2-hydroxypalmitate | **1.39** | 0.0000 | 0.0001 |
| dehydroisoandrosterone sulfate (DHEA-S) | **0.62** | 0.0000 | 0.0001 |
| Leucylleucine | **1.85** | 0.0001 | 0.0002 |
| arachidonate (20:4n6) | **1.57** | 0.0000 | 0.0002 |
| Glycerate | **1.38** | 0.0001 | 0.0003 |
| caproate (6:0) | **0.85** | 0.0001 | 0.0003 |
| Tetradecanedioate | **1.76** | 0.0001 | 0.0004 |
| Isovalerate | **0.75** | 0.0002 | 0.0005 |
| C-glycosyltryptophan | **1.27** | 0.0002 | 0.0006 |
| Theophylline | **10.43** | 0.0002 | 0.0006 |
| ADSGEGDFXAEGGGVR* | **3.96** | 0.0003 | 0.0009 |
| 2-docosahexaenoylglycerophosphoethanolamine* | **1.93** | 0.0004 | 0.0010 |
| Nicotinamide | **0.70** | 0.0004 | 0.0010 |
| epiandrosterone sulfate | **0.57** | 0.0005 | 0.0012 |
| trigonelline (N'-methylnicotinate) | **3.15** | 0.0005 | 0.0012 |
| N-acetylneuraminate | **1.46** | 0.0006 | 0.0013 |
| Cholesterol | **1.17** | 0.0006 | 0.0014 |
| oxalate (ethanedioate) | **2.50** | 0.0007 | 0.0014 |
| gamma-CEHC | **0.61** | 0.0007 | 0.0014 |
| heptanoate (7:0) | **0.85** | 0.0008 | 0.0015 |
| 2-hydroxyisobutyrate | **1.31** | 0.0009 | 0.0016 |
| Mannose | **1.31** | 0.0010 | 0.0018 |
| DSGEGDFXAEGGGVR* | **2.90** | 0.0011 | 0.0020 |
| 1-linoleoylglycerophosphocholine (18:2n6) | **0.79** | 0.0015 | 0.0025 |
| 2-linoleoylglycerophosphocholine* | **0.77** | 0.0014 | 0.0025 |
| Piperine | **0.59** | 0.0014 | 0.0025 |
| Alanine | **0.82** | 0.0016 | 0.0026 |
| glycerophosphorylcholine (GPC) | **1.61** | 0.0020 | 0.0032 |
| Xanthine | **2.49** | 0.0020 | 0.0032 |
| docosahexaenoate (DHA; 22:6n3) | **1.57** | 0.0022 | 0.0034 |
| Methionine | **0.86** | 0.0027 | 0.0041 |
| 3-hydroxybutyrate (BHBA) | **2.55** | 0.0030 | 0.0044 |
| androsterone sulfate | **0.64** | 0.0030 | 0.0044 |
| gamma-tocopherol | **0.74** | 0.0033 | 0.0046 |
| oleate (18:1n9) | **1.49** | 0.0040 | 0.0055 |
| docosapentaenoate (n3 DPA; 22:5n3) | **1.55** | 0.0042 | 0.0057 |
| palmitoleate (16:1n7) | **1.62** | 0.0049 | 0.0066 |
| Tryptophan | **0.89** | 0.0054 | 0.0068 |
| Glycerol | **1.25** | 0.0053 | 0.0068 |
| 4-acetaminophen sulfate | **4.47** | 0.0053 | 0.0068 |
| dihomo-linoleate (20:2n6) | **1.45** | 0.0061 | 0.0075 |
| Indoleacetate | **0.76** | 0.0065 | 0.0077 |
| Creatinine | **0.88** | 0.0067 | 0.0077 |
| Xylonite | **1.41** | 0.0067 | 0.0077 |
| 10-heptadecenoate (17:1n7) | **1.42** | 0.0068 | 0.0077 |
| pregnenolone sulfate | **0.82** | 0.0065 | 0.0077 |
| Valine | **0.91** | 0.0072 | 0.0080 |
| 4-androsten-3beta,17beta-diol disulfate (2) | **0.88** | 0.0076 | 0.0084 |
| 1-pentadecanoylglycerophosphocholine (15:0)* | **0.84** | 0.0081 | 0.0087 |
| Biliverdin | **1.53** | 0.0081 | 0.0087 |
| Isovalerylcarnitine | **0.81** | 0.0093 | 0.0097 |
| alpha-hydroxyisocaproate | **1.44** | 0.0106 | 0.0109 |
| pro-hydroxy-pro | **1.34** | 0.0110 | 0.0112 |
| Cholate | **3.96** | 0.0116 | 0.0117 |
| 1-oleoylglycerophosphocholine (18:1) | **0.82** | 0.0124 | 0.0122 |
| palmitate (16:0) | **1.27** | 0.0130 | 0.0125 |
| 1,6-anhydroglucose | **0.74** | 0.0129 | 0.0125 |
| alpha-ketoglutarate | **1.18** | 0.0133 | 0.0126 |
| 3-methoxytyrosine | **2.84** | 0.0138 | 0.0128 |
| 10-nonadecenoate (19:1n9) | **1.45** | 0.0139 | 0.0128 |
| Creatine | **1.36** | 0.0160 | 0.0146 |
| eicosapentaenoate (EPA; 20:5n3) | **1.53** | 0.0167 | 0.0148 |
| docosapentaenoate (n6 DPA; 22:5n6) | **1.57** | 0.0165 | 0.0148 |
| N-acetylserine | **1.19** | 0.0193 | 0.0169 |
| Pipecolate | **1.44** | 0.0199 | 0.0172 |
| 1-palmitoylglycerophosphocholine (16:0) | **0.88** | 0.0210 | 0.0179 |
| gamma-glutamylglutamine | **1.18** | 0.0230 | 0.0194 |
| pelargonate (9:0) | **0.90** | 0.0241 | 0.0194 |
| myristoleate (14:1n5) | **1.50** | 0.0238 | 0.0194 |
| 2-hydroxystearate | **1.23** | 0.0240 | 0.0194 |
| Hypoxanthine | **1.36** | 0.0233 | 0.0194 |
| adrenate (22:4n6) | **1.42** | 0.0257 | 0.0203 |
| pregn steroid monosulfate* | **0.79** | 0.0258 | 0.0203 |
| Heme | **1.44** | 0.0262 | 0.0204 |
| Isoleucine | **0.88** | 0.0269 | 0.0207 |
| Cortisone | **0.88** | 0.0279 | 0.0212 |
| Leucine | **0.89** | 0.0306 | 0.0228 |
| 2-stearoylglycerophosphocholine* | **0.78** | 0.0306 | 0.0228 |
| 2-myristoylglycerophosphocholine* | **0.88** | 0.0311 | 0.0229 |
| Histidine | **0.85** | 0.0325 | 0.0236 |
| 2-hydroxyglutarate | **1.43** | 0.0331 | 0.0236 |
| Decanoylcarnitine | **1.55** | 0.0334 | 0.0236 |
| Cortisol | **0.88** | 0.0338 | 0.0236 |
| Theobromine | **0.65** | 0.0333 | 0.0236 |
| 2-hydroxybutyrate (AHB) | **1.26** | 0.0372 | 0.0257 |
| Propionylcarnitine | **0.88** | 0.0395 | 0.0271 |
| 4-vinylphenol sulfate | **0.66** | 0.0399 | 0.0271 |
| Hippurate | **1.49** | 0.0406 | 0.0272 |
| Sphingosine | **0.50** | 0.0410 | 0.0273 |
| Pseudouridine | **1.12** | 0.0414 | 0.0273 |
| Hexanoylcarnitine | **1.45** | 0.0421 | 0.0275 |
| Valerate | **1.27** | 0.0432 | 0.0279 |
| 1-palmitoylglycerophosphoethanolamine | **1.27** | 0.0457 | 0.0293 |
| 4-androsten-3beta,17beta-diol disulfate (1) | **1.10** | 0.0504 | 0.0319 |
| 4-acetamidophenol | **6.44** | 0.0507 | 0.0319 |
| 5-dodecenoate (12:1n7) | **1.64** | 0.0527 | 0.0325 |
| pentadecanoate (15:0) | **1.16** | 0.0521 | 0.0325 |
| Lysine | **1.17** | 0.0556 | 0.0337 |
| Proline | **0.88** | 0.0556 | 0.0337 |
| Arabinose | **1.24** | 0.0569 | 0.0339 |
| erythronate* | **1.12** | 0.0563 | 0.0339 |
| arachidate (20:0) | **1.16** | 0.0590 | 0.0348 |
| 2-palmitoylglycerophosphocholine* | **0.84** | 0.0594 | 0.0348 |
| 13-HODE + 9-HODE | **0.88** | 0.0612 | 0.0356 |
| 5alpha-androstan-3beta,17beta-diol disulfate | **1.02** | 0.0667 | 0.0385 |
| 1-margaroylglycerophosphocholine (17:0) | **0.87** | 0.0750 | 0.0429 |
| Indolepropionate | **0.69** | 0.0772 | 0.0435 |
| Succinylcarnitine | **1.16** | 0.0777 | 0.0435 |
| pregnen-diol disulfate* | **0.89** | 0.0780 | 0.0435 |
| Fructose | **1.30** | 0.0798 | 0.0442 |
| alpha-hydroxyisovalerate | **1.23** | 0.0808 | 0.0444 |
| Oleoylcarnitine | **1.29** | 0.0866 | 0.0472 |
| stearate (18:0) | **1.15** | 0.0938 | 0.0507 |
| N1-Methyl-2-pyridone-5-carboxamide | **0.64** | 0.0970 | 0.0520 |

e-Table 2 (Supplemental Material) List of metabolites that differentiate COPD non-survivor discovery (NSd) vs. COPD survivor (S). Results are ranked according to the Welch’s t tests results. Color coded results according to the level of significance (see legend above)

| **Metabolite** | **NSd / S** |  |  |
| --- | --- | --- | --- |
|  |  | ***p*-value** | ***q*-value** |
| Benzoate | **0.60** | 0.0000 | 0.0000 |
| 2-ethylhexanoate | **6.71** | 0.0000 | 0.0000 |
| Fumarate | **1.47** | 0.0000 | 0.0000 |
| Succinate | **1.55** | 0.0000 | 0.0000 |
| caproate (6:0) | **0.79** | 0.0000 | 0.0000 |
| heptanoate (7:0) | **0.76** | 0.0000 | 0.0001 |
| Ornithine | **1.89** | 0.0000 | 0.0001 |
| Malate | **1.78** | 0.0000 | 0.0001 |
| N-acetylneuraminate | **1.70** | 0.0000 | 0.0001 |
| Lactate | **1.30** | 0.0000 | 0.0002 |
| Piperine | **0.51** | 0.0000 | 0.0002 |
| Hexadecanedioate | **2.00** | 0.0000 | 0.0003 |
| alpha-hydroxyisocaproate | **1.77** | 0.0000 | 0.0004 |
| alpha-ketoglutarate | **1.62** | 0.0000 | 0.0004 |
| arachidonate (20:4n6) | **1.58** | 0.0001 | 0.0011 |
| Glycerate | **1.37** | 0.0001 | 0.0012 |
| Lysine | **1.34** | 0.0002 | 0.0014 |
| trigonelline (N'-methylnicotinate) | **3.42** | 0.0002 | 0.0015 |
| glycerophosphorylcholine (GPC) | **1.92** | 0.0002 | 0.0017 |
| Isovalerate | **0.77** | 0.0003 | 0.0022 |
| Fructose | **1.59** | 0.0003 | 0.0022 |
| gamma-CEHC | **0.65** | 0.0005 | 0.0032 |
| 1-arachidonoylglycerophosphoinositol* | **1.40** | 0.0006 | 0.0036 |
| pelargonate (9:0) | **0.83** | 0.0007 | 0.0045 |
| bradykinin, des-arg(9) | **2.17** | 0.0008 | 0.0046 |
| 1,5-anhydroglucitol (1,5-AG) | **0.76** | 0.0009 | 0.0052 |
| Aspartate | **1.70** | 0.0010 | 0.0053 |
| dihomo-linolenate (20:3n3 or n6) | **1.45** | 0.0010 | 0.0055 |
| 2-hydroxyisobutyrate | **1.30** | 0.0011 | 0.0059 |
| 5-oxoproline | **1.16** | 0.0014 | 0.0066 |
| Xylonite | **1.51** | 0.0014 | 0.0066 |
| gamma-tocopherol | **0.63** | 0.0015 | 0.0069 |
| 1-palmitoylglycerol (1-monopalmitin) | **1.49** | 0.0016 | 0.0074 |
| docosahexaenoate (DHA; 22:6n3) | **1.60** | 0.0017 | 0.0075 |
| HWESASXX* | **1.62** | 0.0020 | 0.0083 |
| 2-docosahexaenoylglycerophosphoethanolamine* | **1.79** | 0.0020 | 0.0083 |
| oxalate (ethanedioate) | **2.40** | 0.0022 | 0.0089 |
| C-glycosyltryptophan | **1.23** | 0.0030 | 0.0116 |
| myo-inositol | **1.28** | 0.0036 | 0.0139 |
| 1-linoleoylglycerophosphocholine (18:2n6) | **0.84** | 0.0041 | 0.0153 |
| 2-aminobutyrate | **0.81** | 0.0044 | 0.0158 |
| N-acetyl-beta-alanine | **0.79** | 0.0045 | 0.0158 |
| eicosapentaenoate (EPA; 20:5n3) | **1.64** | 0.0047 | 0.0160 |
| Biliverdin | **1.60** | 0.0047 | 0.0160 |
| ADSGEGDFXAEGGGVR* | **3.23** | 0.0059 | 0.0194 |
| Fucose | **1.48** | 0.0061 | 0.0198 |
| Creatine | **1.43** | 0.0064 | 0.0203 |
| Hippurate | **1.66** | 0.0087 | 0.0269 |
| Leucylleucine | **1.53** | 0.0096 | 0.0293 |
| Glycodeoxycholate | **0.90** | 0.0100 | 0.0297 |
| 4-vinylphenol sulfate | **0.48** | 0.0103 | 0.0301 |
| erythronate* | **1.17** | 0.0111 | 0.0319 |
| DSGEGDFXAEGGGVR* | **2.44** | 0.0129 | 0.0363 |
| Indoleacetate | **0.84** | 0.0137 | 0.0379 |
| gamma-glutamylglutamine | **1.19** | 0.0145 | 0.0384 |
| 13-HODE + 9-HODE | **0.83** | 0.0145 | 0.0384 |
| Glycerol | **1.37** | 0.0147 | 0.0384 |
| N-acetylserine | **1.20** | 0.0152 | 0.0390 |
| 2-hydroxyglutarate | **1.49** | 0.0165 | 0.0416 |
| Threitol | **1.30** | 0.0171 | 0.0423 |
| 2-hydroxypalmitate | **1.23** | 0.0177 | 0.0423 |
| Xanthine | **1.91** | 0.0178 | 0.0423 |
| Nicotinamide | **0.71** | 0.0179 | 0.0423 |
| Betaine | **0.87** | 0.0190 | 0.0443 |
| gamma-glutamylisoleucine* | **1.27** | 0.0200 | 0.0458 |
| cis-vaccenate (18:1n7) | **0.74** | 0.0216 | 0.0488 |
| cis-4-decenoyl carnitine | **0.82** | 0.0232 | 0.0509 |
| 1-pentadecanoylglycerophosphocholine (15:0)* | **0.80** | 0.0232 | 0.0509 |
| Tetradecanedioate | **1.33** | 0.0265 | 0.0573 |
| Sphingosine | **1.27** | 0.0289 | 0.0615 |
| Threonate | **0.70** | 0.0296 | 0.0622 |
| Theobromine | **0.67** | 0.0314 | 0.0650 |
| 1-palmitoylglycerophosphoethanolamine | **1.30** | 0.0331 | 0.0669 |
| Erythritol | **1.22** | 0.0332 | 0.0669 |
| 2-stearoylglycerophosphocholine* | **0.83** | 0.0369 | 0.0733 |
| pro-hydroxy-pro | **1.27** | 0.0378 | 0.0741 |
| Arabitol | **1.27** | 0.0424 | 0.0816 |
| 10-undecenoate (11:1n1) | **0.89** | 0.0427 | 0.0816 |
| Pipecolate | **1.39** | 0.0481 | 0.0907 |
| 2-linoleoylglycerophosphocholine* | **0.87** | 0.0567 | 0.1055 |
| 2-hydroxy-3-methylvalerate | **1.49** | 0.0597 | 0.1098 |
| Stearoylcarnitine | **0.85** | 0.0612 | 0.1112 |
| Cholesterol | **1.09** | 0.0624 | 0.1120 |
| glycolate (hydroxyacetate) | **1.09** | 0.0658 | 0.1168 |
| docosapentaenoate (n3 DPA; 22:5n3) | **1.35** | 0.0708 | 0.1242 |
| 1-docosahexaenoylglycerophosphocholine (22:6n3)* | **1.27** | 0.0738 | 0.1279 |
| N1-Methyl-2-pyridone-5-carboxamide | **0.88** | 0.0805 | 0.1379 |
| gamma-glutamylleucine | **1.15** | 0.0909 | 0.1539 |

e-Table 3 (Supplemental Material) List of metabolites that differentiate COPD survivor (S) vs. Control (C). Results are ranked according to the Welch’s t tests results. Color coded results according to the level of significance (see legend above)

| **Metabolite** | **S / C** | **p value** | **q value** |
| --- | --- | --- | --- |
| Leucine | **0.84** | 0.0039 | 0.1529 |
| Isoleucine | **0.83** | 0.0046 | 0.1529 |
| Valine | **0.88** | 0.0028 | 0.1529 |
| Methionine | **0.84** | 0.0024 | 0.1529 |
| gamma-glutamylleucine | **0.80** | 0.0035 | 0.1529 |
| pregnenolone sulfate | **0.72** | 0.0042 | 0.1529 |
| dehydroisoandrosterone sulfate (DHEA-S) | **0.71** | 0.0024 | 0.1529 |
| androsterone sulfate | **0.65** | 0.0066 | 0.1918 |
| Tryptophan | **0.87** | 0.0104 | 0.2019 |
| Mannose | **1.19** | 0.0096 | 0.2019 |
| epiandrosterone sulfate | **0.68** | 0.0089 | 0.2019 |
| 4-androsten-3beta,17beta-diol disulfate (2) | **0.74** | 0.0082 | 0.2019 |
| Cortisol | **0.76** | 0.0114 | 0.2043 |
| HWESASXX* | **1.38** | 0.0144 | 0.2396 |
| 5-dodecenoate (12:1n7) | **1.55** | 0.0174 | 0.2463 |
| Cortisone | **0.81** | 0.0164 | 0.2463 |
| 4-androsten-3beta,17beta-diol disulfate (1) | **0.74** | 0.0180 | 0.2463 |
| Lysine | **0.87** | 0.0236 | 0.2959 |
| 4-ethylphenylsulfate | **0.45** | 0.0242 | 0.2959 |
| 3-hydroxybutyrate (BHBA) | **1.79** | 0.0295 | 0.3421 |
| Alanine | **0.87** | 0.0470 | 0.4162 |
| gamma-glutamylvaline | **0.86** | 0.0408 | 0.4162 |
| Fucose | **1.33** | 0.0441 | 0.4162 |
| myristoleate (14:1n5) | **1.40** | 0.0463 | 0.4162 |
| Carnitine | **0.92** | 0.0465 | 0.4162 |
| pregnen-diol disulfate* | **0.78** | 0.0441 | 0.4162 |
| 4-acetaminophen sulfate | **2.77** | 0.0484 | 0.4162 |
| oleate (18:1n9) | **1.34** | 0.0521 | 0.4169 |
| Theophylline | **7.16** | 0.0515 | 0.4169 |
| Phenylalanine | **0.93** | 0.0587 | 0.4260 |
| 3-methoxytyrosine | **1.14** | 0.0562 | 0.4260 |
| Fructose | **0.82** | 0.0599 | 0.4260 |
| 10-nonadecenoate (19:1n9) | **1.38** | 0.0605 | 0.4260 |
| 3-hydroxydecanoate | **1.19** | 0.0640 | 0.4374 |
| 5alpha-androstan-3beta,17beta-diol disulfate | **0.84** | 0.0682 | 0.4526 |
| alpha-hydroxyisocaproate | **0.81** | 0.0844 | 0.4664 |
| Proline | **0.87** | 0.0767 | 0.4664 |
| N-acetylneuraminate | **0.86** | 0.0786 | 0.4664 |
| palmitate (16:0) | **1.21** | 0.0728 | 0.4664 |
| palmitoleate (16:1n7) | **1.38** | 0.0835 | 0.4664 |
| Propionylcarnitine | **0.87** | 0.0801 | 0.4664 |
| 4-acetamidophenol | **2.98** | 0.0805 | 0.4664 |
| tryptophan betaine | **1.32** | 0.0915 | 0.4723 |
| heptanoate (7:0) | **1.11** | 0.0911 | 0.4723 |
| pentadecanoate (15:0) | **1.11** | 0.0886 | 0.4723 |

e-Table 4 (Supplemental Material ). List of metabolites that are different between the 3 groups by two sample t-test, linear discriminate analysis (LDA) and SVM-RFE. NSd (COPD Non-Survivors discovery), S (COPD survivors) and C (Controls)

| NSd vs. S | | |
| --- | --- | --- |
| Sensitivity | specificity | Accuracy |
| 0.7931 | 0.7742 | 0.7833 |
| **'BIOCHEMICAL'** | **Feat_ind** | **Freq_vals** |
| 'cis-aconitate' | 161 | 0.9 |
| 'fumarate' | 201 | 0.9 |
| 'glycolate (hydroxyacetate)' | 228 | 0.9 |
| '1-stearoylglycerol (1-monostearin)' | 31 | 0.7 |
| 'palmitoyl sphingomyelin' | 312 | 0.7 |
| 'pantothenate' | 314 | 0.7 |
| 'succinate' | 362 | 0.7 |
| '1-eicosatrienoylglycerophosphocholine*' | 14 | 0.6 |
| '2-oleoylglycerophosphocholine*' | 59 | 0.6 |
| '2-palmitoylglycerophosphocholine*' | 60 | 0.6 |
| '7-alpha-hydroxy-3-oxo-4-cholestenoate (7-Hoca)' | 98 | 0.6 |
| 'acetylphosphate' | 102 | 0.6 |
| 'fructose' | 199 | 0.6 |
| 'inositol 1-phosphate (I1P)' | 249 | 0.6 |
| 'phosphate' | 327 | 0.6 |
| '1-heptadecanoylglycerophosphocholine' | 15 | 0.5 |
| '1-oleoylglycerophosphocholine' | 23 | 0.5 |
| '1-palmitoylglycerophosphocholine' | 27 | 0.5 |
| '10-undecenoate (11:1n1)' | 36 | 0.5 |
| '2-hydroxypalmitate' | 51 | 0.5 |
| 'erythritol' | 193 | 0.5 |
| 'glycerate' | 218 | 0.5 |
| 'glycerol' | 219 | 0.5 |
| 'pro-hydroxy-pro' | 333 | 0.5 |
| 'scyllo-inositol' | 353 | 0.5 |
| 'stearoyl sphingomyelin' | 360 | 0.5 |
| NSd vs. C | | |
| Sensitivity | specificity | Accuracy |
| 0.8 | 0.7742 | 0.7857 |
| **'BIOCHEMICAL'** | **Feat_ind** | **Freq Vals** |
| 'palmitoyl sphingomyelin' | 312 | 1 |
| 'dehydroisoandrosterone sulfate (DHEA-S)' | 176 | 0.9 |
| 'phosphate' | 327 | 0.9 |
| 'hexadecanedioate' | 235 | 0.8 |
| 'gamma-glutamylalanine' | 204 | 0.7 |
| 'stearoyl sphingomyelin' | 360 | 0.7 |
| 'tetradecanedioate' | 371 | 0.7 |
| '10-heptadecenoate (17:1n7)' | 34 | 0.6 |
| '2-hydroxybutyrate (AHB)' | 46 | 0.6 |
| 'acetylphosphate' | 102 | 0.6 |
| 'gamma-glutamylvaline' | 211 | 0.6 |
| 'inositol 1-phosphate (I1P)' | 249 | 0.6 |
| S vs. C | | |
| Sensitivity | specificity | Accuracy |
| 0.8 | 0.6552 | 0.7333 |
| **'BIOCHEMICAL'** | **Feat ind** | **Freq Vals** |
| 'methionine' | 273 | 0.7 |
| '1-palmitoylglycerol (1-monopalmitin)' | 26 | 0.6 |
| '1-docosahexaenoylglycerophosphocholine*' | 11 | 0.5 |
| '1-eicosatrienoylglycerophosphocholine*' | 14 | 0.5 |
| '1-heptadecanoylglycerophosphocholine' | 15 | 0.5 |
| '1-stearoylglycerol (1-monostearin)' | 31 | 0.5 |
| 'leucine' | 261 | 0.5 |
| 'p-cresol sulfate' | 308 | 0.5 |
| 'valine' | 392 | 0.5 |
| '1-eicosadienoylglycerophosphocholine*' | 13 | 0.4 |
| '2-palmitoylglycerophosphocholine*' | 60 | 0.4 |
| '4-methyl-2-oxopentanoate' | 89 | 0.4 |
| 'docosahexaenoate (DHA; 22:6n3)' | 184 | 0.4 |
| 'eicosapentaenoate (EPA; 20:5n3)' | 190 | 0.4 |
| 'gamma-glutamylalanine' | 204 | 0.4 |
| 'kynurenine' | 255 | 0.4 |
| 'phenylacetylglutamine' | 321 | 0.4 |
| 'scyllo-inositol' | 353 | 0.4 |
| 'threitol' | 374 | 0.4 |

e-Table 5 (Supplemental Material).List of altered metabolic pathways between the group of COPD non survivors discovery (NSd) and COPD survivors (S).The pathway analysis was performed with MetaboAnalyst 3.0 [4]. Please see corresponding figure 3 in the main manuscript.

| Pathway Name | Match Status | p | -log(p) | Holm p | FDR | Impact | Details |
| --- | --- | --- | --- | --- | --- | --- | --- |
| [Glyoxylate and dicarboxylate metabolism](https://www.metaboanalyst.ca/MetaboAnalyst/faces/Secure/pathway/ResultView.xhtml) | [4/50](https://www.metaboanalyst.ca/MetaboAnalyst/faces/Secure/pathway/ResultView.xhtml) | 1.902E-4 | 8.5674 | 0.015216 | 0.0083855 | 0.04294 | [KEGG](http://www.genome.jp/kegg-bin/show_pathway?hsa00630) |
| [Citrate cycle (TCA cycle)](https://www.metaboanalyst.ca/MetaboAnalyst/faces/Secure/pathway/ResultView.xhtml) | [3/20](https://www.metaboanalyst.ca/MetaboAnalyst/faces/Secure/pathway/ResultView.xhtml) | 2.0964E-4 | 8.4701 | 0.016561 | 0.0083855 | 0.08641 | [KEGG](http://www.genome.jp/kegg-bin/show_pathway?hsa00020) [SMP](http://www.smpdb.ca/view/SMP00057) |
| [Alanine, aspartate and glutamate metabolism](https://www.metaboanalyst.ca/MetaboAnalyst/faces/Secure/pathway/ResultView.xhtml) | [2/24](https://www.metaboanalyst.ca/MetaboAnalyst/faces/Secure/pathway/ResultView.xhtml) | 0.0092452 | 4.6836 | 0.72113 | 0.24654 | 0.00285 | [KEGG](http://www.genome.jp/kegg-bin/show_pathway?hsa00250) [SMP](http://www.smpdb.ca/view/SMP00067) [SMP](http://www.smpdb.ca/view/SMP00055) [SMP](http://www.smpdb.ca/view/SMP00072) |
| [Glycerolipid metabolism](https://www.metaboanalyst.ca/MetaboAnalyst/faces/Secure/pathway/ResultView.xhtml) | [2/32](https://www.metaboanalyst.ca/MetaboAnalyst/faces/Secure/pathway/ResultView.xhtml) | 0.016143 | 4.1262 | 1.0 | 0.32287 | 0.20907 | [KEGG](http://www.genome.jp/kegg-bin/show_pathway?hsa00561) [SMP](http://www.smpdb.ca/view/SMP00039) |
| [Butanoate metabolism](https://www.metaboanalyst.ca/MetaboAnalyst/faces/Secure/pathway/ResultView.xhtml) | [2/40](https://www.metaboanalyst.ca/MetaboAnalyst/faces/Secure/pathway/ResultView.xhtml) | 0.024668 | 3.7023 | 1.0 | 0.39468 | 0.03545 | [KEGG](http://www.genome.jp/kegg-bin/show_pathway?hsa00650) [SMP](http://www.smpdb.ca/view/SMP00073) |
| [Phenylalanine metabolism](https://www.metaboanalyst.ca/MetaboAnalyst/faces/Secure/pathway/ResultView.xhtml) | [2/45](https://www.metaboanalyst.ca/MetaboAnalyst/faces/Secure/pathway/ResultView.xhtml) | 0.030752 | 3.4818 | 1.0 | 0.41003 | 0.0 | [KEGG](http://www.genome.jp/kegg-bin/show_pathway?hsa00360) [SMP](http://www.smpdb.ca/view/SMP00008) |
| [Tyrosine metabolism](https://www.metaboanalyst.ca/MetaboAnalyst/faces/Secure/pathway/ResultView.xhtml) | [2/76](https://www.metaboanalyst.ca/MetaboAnalyst/faces/Secure/pathway/ResultView.xhtml) | 0.079241 | 2.5353 | 1.0 | 0.90561 | 0.0 | [KEGG](http://www.genome.jp/kegg-bin/show_pathway?hsa00350) [SMP](http://www.smpdb.ca/view/SMP00006) [SMP](http://www.smpdb.ca/view/SMP00008) |
| [Taurine and hypotaurine metabolism](https://www.metaboanalyst.ca/MetaboAnalyst/faces/Secure/pathway/ResultView.xhtml) | [1/20](https://www.metaboanalyst.ca/MetaboAnalyst/faces/Secure/pathway/ResultView.xhtml) | 0.11797 | 2.1374 | 1.0 | 1.0 | 0.11151 | [KEGG](http://www.genome.jp/kegg-bin/show_pathway?hsa00430) [SMP](http://www.smpdb.ca/view/SMP00021) |
| [Pantothenate and CoA biosynthesis](https://www.metaboanalyst.ca/MetaboAnalyst/faces/Secure/pathway/ResultView.xhtml) | [1/27](https://www.metaboanalyst.ca/MetaboAnalyst/faces/Secure/pathway/ResultView.xhtml) | 0.15609 | 1.8573 | 1.0 | 1.0 | 0.18014 | [KEGG](http://www.genome.jp/kegg-bin/show_pathway?hsa00770) [SMP](http://www.smpdb.ca/view/SMP00027) |
| [beta-Alanine metabolism](https://www.metaboanalyst.ca/MetaboAnalyst/faces/Secure/pathway/ResultView.xhtml) | [1/28](https://www.metaboanalyst.ca/MetaboAnalyst/faces/Secure/pathway/ResultView.xhtml) | 0.16141 | 1.8238 | 1.0 | 1.0 | 0.0 | [KEGG](http://www.genome.jp/kegg-bin/show_pathway?hsa00410) [SMP](http://www.smpdb.ca/view/SMP00055) |
| [Pyruvate metabolism](https://www.metaboanalyst.ca/MetaboAnalyst/faces/Secure/pathway/ResultView.xhtml) | [1/32](https://www.metaboanalyst.ca/MetaboAnalyst/faces/Secure/pathway/ResultView.xhtml) | 0.18237 | 1.7017 | 1.0 | 1.0 | 0.0 | [KEGG](http://www.genome.jp/kegg-bin/show_pathway?hsa00620) [SMP](http://www.smpdb.ca/view/SMP00060) |
| [Pentose phosphate pathway](https://www.metaboanalyst.ca/MetaboAnalyst/faces/Secure/pathway/ResultView.xhtml) | [1/32](https://www.metaboanalyst.ca/MetaboAnalyst/faces/Secure/pathway/ResultView.xhtml) | 0.18237 | 1.7017 | 1.0 | 1.0 | 0.02181 | [KEGG](http://www.genome.jp/kegg-bin/show_pathway?hsa00030) [SMP](http://www.smpdb.ca/view/SMP00031) |
| [Propanoate metabolism](https://www.metaboanalyst.ca/MetaboAnalyst/faces/Secure/pathway/ResultView.xhtml) | [1/35](https://www.metaboanalyst.ca/MetaboAnalyst/faces/Secure/pathway/ResultView.xhtml) | 0.19777 | 1.6206 | 1.0 | 1.0 | 0.00134 | [KEGG](http://www.genome.jp/kegg-bin/show_pathway?hsa00640) [SMP](http://www.smpdb.ca/view/SMP00016) |
| [Glycerophospholipid metabolism](https://www.metaboanalyst.ca/MetaboAnalyst/faces/Secure/pathway/ResultView.xhtml) | [1/39](https://www.metaboanalyst.ca/MetaboAnalyst/faces/Secure/pathway/ResultView.xhtml) | 0.21788 | 1.5238 | 1.0 | 1.0 | 0.00317 | [KEGG](http://www.genome.jp/kegg-bin/show_pathway?hsa00564) |
| [Inositol phosphate metabolism](https://www.metaboanalyst.ca/MetaboAnalyst/faces/Secure/pathway/ResultView.xhtml) | [1/39](https://www.metaboanalyst.ca/MetaboAnalyst/faces/Secure/pathway/ResultView.xhtml) | 0.21788 | 1.5238 | 1.0 | 1.0 | 0.0441 | [KEGG](http://www.genome.jp/kegg-bin/show_pathway?hsa00562) [SMP](http://www.smpdb.ca/view/SMP00011) |
| [Galactose metabolism](https://www.metaboanalyst.ca/MetaboAnalyst/faces/Secure/pathway/ResultView.xhtml) | [1/41](https://www.metaboanalyst.ca/MetaboAnalyst/faces/Secure/pathway/ResultView.xhtml) | 0.22776 | 1.4794 | 1.0 | 1.0 | 0.0 | [KEGG](http://www.genome.jp/kegg-bin/show_pathway?hsa00052) [SMP](http://www.smpdb.ca/view/SMP00043) |
| [Nicotinate and nicotinamide metabolism](https://www.metaboanalyst.ca/MetaboAnalyst/faces/Secure/pathway/ResultView.xhtml) | [1/44](https://www.metaboanalyst.ca/MetaboAnalyst/faces/Secure/pathway/ResultView.xhtml) | 0.24236 | 1.4173 | 1.0 | 1.0 | 0.0 | [KEGG](http://www.genome.jp/kegg-bin/show_pathway?hsa00760) [SMP](http://www.smpdb.ca/view/SMP00048) |

Refereces:

1. Langley RJ, Tsalik EL, van Velkinburgh JC, Glickman SW, Rice BJ, Wang C, et al. An integrated clinico-metabolomic model improves prediction of death in sepsis. Sci Transl Med [Internet]. 2013;5:195ra95. Available from: http://www.ncbi.nlm.nih.gov/pubmed/23884467%5Cnhttp://www.pubmedcentral.nih.gov/articlerender.fcgi?artid=PMC3924586

2. Chen T, Cao Y, Zhang Y, Liu J, Bao Y, Wang C, et al. Random forest in clinical metabolomics for phenotypic discrimination and biomarker selection. Evidence-based Complement Altern Med. 2013;2013.

3. Byvatov E, Schneider G. Support vector machine applications in bioinformatics. Appl Bioinformatics. 2003;2:67–77.

4. Xia J, Sinelnikov I V., Han B, Wishart DS. MetaboAnalyst 3.0-making metabolomics more meaningful. Nucleic Acids Res. 2015;
